# Emergency Sonography Aids Diagnostic Accuracy of Torso Injuries: A Study in a Resource Limited Setting

**DOI:** 10.1155/2014/978795

**Published:** 2014-07-08

**Authors:** Charles Edward Tunuka, Robert Wangoda, Sam Bugeza, Moses Galukande

**Affiliations:** ^1^Department of Surgery, Makerere University College of Health Sciences, Kampala, Uganda; ^2^Department of Radiology, Makerere University College of Health Sciences, Kampala, Uganda

## Abstract

*Introduction*. Clinical evaluation of patients with torso trauma is often a diagnostic challenge. Extended focused assessment with sonography for trauma (EFAST) is an emergency ultrasound scan that adds to the evaluation of intrathoracic abdominal and pericardial cavities done in FAST (focused assessment with sonography for trauma). *Objective*. This study compares EFAST (the index test) with the routine standard of care (SoC) investigations (the standard reference test) for torso trauma injuries. *Methods*. A cross-sectional descriptive study was conducted over a 3-month period. Eligible patients underwent EFAST scanning and the SoC assessment. The diagnostic accuracy of EFAST was calculated using sensitivity and specificity scores. *Results*. We recruited 197 patients; the M : F ratio was 5 : 1, with mean age of 27 years (SD 11). The sensitivity of EFAST was 100%, the specificity was 97%, the PPV was 87%, and the NPV was 100%. It took 5 minutes on average to complete an EFAST scan. 168 (85%) patients were EFAST-scanned. Most patients (82) (48%) were discharged on the same day of hospitalization, while 7 (4%) were still at the hospital after two weeks. The mortality rate was 18 (9%). *Conclusion*. EFAST is a reliable method of diagnosing torso injuries in a resource limited context.

## 1. Introduction

Evaluation of patients with torso trauma is often a diagnostic challenge for emergency physicians and trauma surgeons. Uncontrolled hemorrhage is responsible for over 50% of trauma related deaths [1–3]. Significant bleeding into the peritoneal, pleural, or pericardial spaces may occur without obvious signs [4, 5]. Physical findings may be unreliable because of decreased patient consciousness, neurologic deficit, medication, or other associated injuries like fractures of lower chest ribs, contusion, and abrasions of the abdominal wall. All these call for a need to confirm internal injury by imaging as uncontrolled haemorrhage because torso trauma is one of the major causes of early trauma deaths [6].

However, in many rich trauma centers, bedside ultrasound is the initial imaging modality used to evaluate patients with blunt and penetrating torso trauma. This cannot be said for many countries in sub-Saharan Africa. Yet bedside ultrasound is cheap, noninvasive, and fast leading to early intervention and hence potential reduction in mortality [5, 7].

The purpose of this study therefore was to determine the diagnostic accuracy of EFAST when compared with the routine standard of care (SoC) assessment for torso trauma injuries in a resource limited setting.

## 2. Methods

### 2.1. Design

A cross-sectional analytical study was conducted.

#### 2.1.1. Study Setting

The study was carried out at the A&E unit of Mulago National Referral and Teaching Hospital in Kampala city. The unit is fully fledged with medical and surgical wings, two operating rooms, with X-ray and ultrasound facilities, a high dependence unit (with three beds), and a thirty-bed holding emergency ward. Adjacent to it are blood bank, hematology, microbiology, and clinical chemistry laboratories. The unit sees on average 30 patients with internal torso per month.

#### 2.1.2. Study Population

All patients who were clinically suspected to have torso injury presented to the A&E unit in Mulago Hospital during the study period and consented to the study.

These included patients with torso abrasions and/or bruises, unconsciousness, multiple injuries, alcohol intoxication with trauma, long bone fractures, pelvic fractures, and spine injuries.

Those with penetrating injuries and burns with no other trauma injuries were excluded.

### 2.2. Sampling

Patients were recruited consecutively on arrival at the A&E unit; they were triaged and transferred to the appropriate examination rooms for further assessment according to the ATLS protocol. Patients in need of operative management were immediately taken to the operating room while those assigned to the nonoperative management plan were admitted to the 24 h holding emergency ward for observation.

Patients consented after they were resuscitated; a special request was obtained from the IRB for a waiver of consent for the unconscious and those without relatives. History and examination findings were recorded on precoded questionnaires. Patients suspected to have torso injuries underwent EFAST using SonoSite TITAN portable ultrasound machine with a transducer frequency ranging from 3.5 to 5 MHz. Images were saved to be reread by a consultant radiologist as a quality control measure. Patients then underwent secondary survey followed by other routine investigations and management according to the hospital's SoC; this consisted of a CXR and abdominal ultrasound scanning preceded by a physical examination and history taking. The CXR was taken by a radiographer and read by an experienced radiologist. The abdominal ultrasound scans were taken by experienced operators. The history taking and physical examinations were performed by an intern doctor and validated by residents and a consultant surgeon in succession.

### 2.3. EFAST Procedure

#### 2.3.1. Examination of the Right Upper Quadrant

The transducer was placed in the midaxillary line between the 11th and 12th ribs, applying coronal scan with the probe (cranially or caudally and medially or laterally) to obtain an optimal image of Morison's pouch and looking for free fluid in it. Findings were recorded on coded questionnaires.

#### 2.3.2. Assessing for Fluid in Right Pleural Cavity

The probe was moved slightly upwards from position of Morison's pouch to look for fluid in the right pleural cavity. Findings were recorded on a coded questionnaire.

#### 2.3.3. Examination of the Left Upper Quadrant

The probe was placed along the left posterior axillary line between the 8th and 11th ribs. If rib shadows were seen then the probe was placed between the ribs (along the intercostal space) to avoid poor acoustic window. Findings were recorded on coded questionnaires.

#### 2.3.4. Assessing for Fluid in Left Pleural Cavity

The probe from splenorenal view was angled with beam direction more cephalad for well visualisation of the spleen and diaphragm and the fluid above the diaphragm was observed. Findings were recorded on coded questionnaires.

#### 2.3.5. Assessing for Free Fluid in Pelvis

The probe was placed in transverse position 2 cm above pubis (for transverse imaging of the bladder) and then turned longitudinally (for longitudinal imaging of the bladder). Findings were recorded on coded questionnaires.

#### 2.3.6. Assessing for Pneumothorax

The probe was placed perpendicular to the ribs in the anterior chest region intercostal spaces 2-3 along the midclavicular line. This was usually done at 3rd-4th intercostal spaces. When visualization was inadequate, the probe was rotated on 90 degrees, placing it directly in the intercostal space along the ribs. Absence of “lung sliding” was a sign of pneumothorax. Findings were recorded on coded questionnaires.

#### 2.3.7. Assessing for Fluid in Pericardium

Subxiphoid pericardial window which has been considered the gold standard for the diagnosis of pericardial effusion was used. In the positive examination there was anechoic space (collection of fluid) between the heart and the pericardium. Findings were recorded on a coded questionnaire.

### 2.4. Study Variables

These included age, sex, tribe, and occupation.

In addition, signs indicating torso trauma were abrasions and bruises. Unconsciousness, multiple injuries, alcohol intoxication, long bone fractures, pelvic fractures, and spine injuries were the other variables.

Hemoperitoneum, hemopericardium, hemothorax, and pneumothorax were also included.

### 2.5. Data Collection, Management, and Analysis

Data collected using pretested questionnaires were double entered, coded, and cleaned using EpiData version 5.3.2 software package.

Stored data were exported to STATA version 12 for analysis. Categorical and numerical variables were summarized using portions, frequency tables, pie charts, and bar charts. Continuous data were summarized into means, medians, and standard deviations. Usefulness of EFAST was determined by calculating sensitivity and specificity of EFAST diagnoses after comparison with the SoC diagnoses made.

### 2.6. Ethical Considerations

Written informed consent was obtained from the participants and permission was obtained from IRB for the patients unable to consent because of their unconscious state and having no available next-of-kin.

## 3. Results

**Figure 1:**
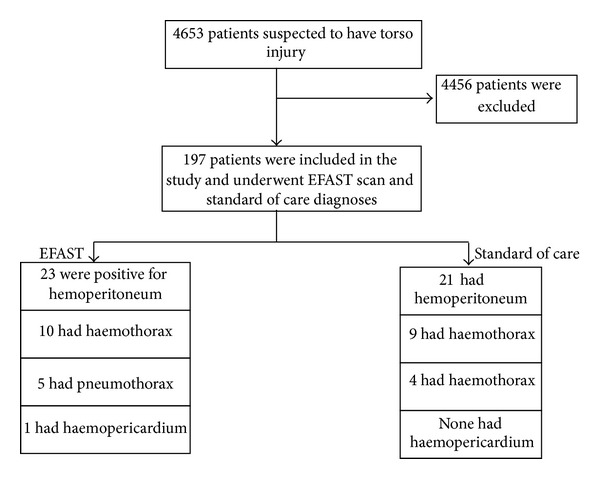
Study for the emergency ultrasound scan torso trauma Uganda study, 2012.

A total of 197 patients were clinically suspected to have torso injury. These patients were subjected to EFAST and the SoC. EFAST scanning took on average 5 minutes to complete for each patient. The scanning was done during resuscitation just after the primary survey. The study was done from January to March 2012 (see Figure 1).

### 3.1. The Baseline Characteristics

The male : female ratio was 5 : 1. The age ranged from 2 to 83 years with mean age 26.9 years (SD of 11). There were 29 (14%) victims whose age was less than 18 years, 158 (80%) were aged between 19 and 45 years, and 10 were >45 years old. 78 (40%) patients arrived at unit before an hour had elapsed from time of injury. 168 (85%) patients were EFAST scanned within an hour from admission while most patients (105) (54%) underwent SoC investigations after an hour of stay at the hospital. Most patients (82) (48%) were discharged on the same day of hospitalization. By the end of the first week 151 (77%) patients had been discharged while 13 (7%) patients were discharged on the second week and 7 (4%) were still at the hospital by the end of the two weeks. 18 (9%) of the participants had died (see Table 1).

Most patients (72%) had undergone EFAST within 30 minutes of admission.

In one hour the emergency room doctor could establish a diagnosis on internal injury in only 17% of patients.

RTC was the main reason for hospitalization.

### 3.2. Circumstances of Injury

The cause of most of the injuries was due to motor traffic crashes (127) (65%). Assault was the second largest cause of injuries affecting 61 patients (31%), followed by falls affecting 6 individuals (3%), while other causes contributed to 3 injuries (2%). Of the motor traffic crashes 53 were the most affected (27%) followed by motor bike victims (41) (21%). Taxis and private cars contributed to the remaining percentage of the victims. Of the motorbike injuries 31 (62%) were the motorbike riders while 19 (38%) were passengers.

### 3.3. External Injuries

There were 23 (12%) participants who did not present with any external injuries. In most instances, participants had more than one presentation of external injuries. All presentations were recognized at the time of admission and the sites of external injuries (see Table 2).

Most of the patients (168) (85%) were bruised and one was burnt (0.50%).

The chest was the most affected region in 130 (66%) followed by the head.

### 3.4. EFAST Findings

Of 197 patients who underwent EFAST, 23 (12%) were positive for hemoperitoneum, 10 (5%) were positive for hemothorax, 5 (2.5%) were positive for pneumothorax, and one (0.5%) was positive for hemopericardium.

### 3.5. SoC Findings

Among patients who underwent standard of care investigations there were 21 (11%) patients with hemoperitoneum, 9 (5%) were positive for hemothorax, 4 (2%) were positive for pneumothorax, and none was positive for hemopericardium.

### 3.6. Validity Testing

Validity testing was done as shown in Table 3.

### 3.7. Management Strategies

Overall 14 (7%) patients were managed operatively while 83 (93%) were managed nonoperatively. There were 4 (2%) patients with hemoperitoneum and 8 (4%) with hemothorax. One patient had a grade four splenic injury and one other a liver laceration in combination with gut perforation. One patient who failed nonoperative management with a hemoperitoneum died en route to the operating room and the eight patients with hemothoraces had tube thoracostomies done.

## 4. Discussion

We set out to determine the utility of emergency ultrasound scanning for the torso trauma patients in a resource limited context. We found that EFAST had high sensitivity and specificity in a low-resourced, high patient turnover environment. There were more males than females injured, similar to a previous study at the unit [8]. The mean age was 27 years attesting to the fact that the youths are the most vulnerable [8, 9].

A fair number of patients (78) (40%) arrived at the A&E within the first hour of injury. This applies more to patients whose district of origin was within 25 km of radius of the hospital. Most of the patients who came from more peripheral districts arrived at the hospital by the second day.

Most of the patients underwent EFAST within 30 minutes of admission; 159 (81%) had undergone EFAST within one hour. Most of the SoC diagnoses did not differ from those reached by bedside ultrasound (EFAST). Several studies support bedside ultrasound as it is fast, affordable, and noninvasive [10–12]. The concept of the golden hour in trauma stresses the fact that most trauma patients can be saved if attended immediately after injury. This can be helped if an ultrasound machine is stationed within the resuscitation room and performed immediately when internal injury is suspected during initial assessment [5, 7]. EFAST improves care by providing a quicker method of assessment, less reliance of radiology staff (who we have few of), and by its ready availability within the resuscitation room.

### 4.1. EFAST and Standard of Care

#### 4.1.1. Hemoperitoneum

In patients who underwent both EFAST and SoC which included full abdominal ultrasound scanning, 23 (12%) were positive at EFAST and 21 were positive at full abdominal ultrasound scanning. The two patients who were considered positive at EFAST but negative when subjected to SoC investigations were among the nonoperatively managed group; therefore EFAST findings could not be validated. Two patients who were considered positive when subjected to SoC investigations underwent surgery and a specific diagnosis of solid organ injury was made. In these two patients the radiological diagnosis was ruptured spleen; however, a ruptured liver and urinary bladder were missed in the first patient and a ruptured spleen was missed in the second patient. Several studies point out that ultrasound is very sensitive in fluid detection but poorly sensitive in solid organ injuries detection [5].

In one patient both EFAST and abdominal ultrasound scan diagnoses were normal but on the second day the patient was still complaining of severe abdominal pain and the abdomen was distended. An exploratory laparotomy was done and the patient had a rupture small gut. Ultrasound is shown by several studies to be sensitive for detection of abdominal fluid accumulation, which is assumed to be blood in acute trauma but has little role in diagnosis of gut perforation in which it can describe fluid level in nonacute perforation [5, 6].

In two other patients, ruptured small gut was missed by both EFAST and full abdominal ultrasound scans only to be diagnosed intraoperatively on failure of conservative management.

#### 4.1.2. Hemothorax

Of the patients who underwent both EFAST and standard of care, chest X-ray in this case, for thoracic complaints 10 (5%) were positive on EFAST and 9 (5%) on chest X-ray. The entire nine patients were managed according to the standard of care. The patient who was positive on EFAST alone was managed nonoperatively because the hemothorax was small. Ultrasound is more sensitive in the detection of hemothorax than a chest X-ray [5, 13].

Ultrasound can detect as little as less than 20 milliliters while an ordinary chest X-ray can pick a hemothorax of 150 mLs on maneuvering the position of the patient when taking the X-ray picture [5].

#### 4.1.3. Pneumothorax

Five (3%) and 4 patients (2%) were positive for pneumothorax on EFAST and SoC, respectively. (In SoC a chest X-ray was used.) All patients were managed operatively. Thoracic ultrasound is shown by several studies to be highly sensitive in detecting hemopneumothorax. Its sensitivity has been demonstrated to be up to 100% and specificity of 99.7% [5]. This is reflected in this study in which one patient diagnosis was missed in the initial chest X-ray (standard of care).

#### 4.1.4. Hemopericardium

There was one patient who had a hemopericardium detected as a small collection on ultrasound but was missed on chest X-ray. This may not have been blood but could be an effusion. The patient was managed conservatively. As it is shown by many other previous studies, ultrasound is very sensitive in fluid detection [10].

#### 4.1.5. EFAST Performance

Performance of EFAST was assessed by calculating diagnostic sensitivity, specificity, and positive and negative predictive values.

EFAST sensitivity and specificity were 100% and 97%, respectively. Positive and negative predictive values were 87% and 100%, respectively. The findings are similar to several other studies [5, 14–16].

#### 4.1.6. Patients' Deposition at Two Weeks

Patients were followed up for two weeks. For patients who had been discharged a phone call was made at the end of the second week to get general information on how they are and if they got back to any hospital for a reason of worsening of the discharge diagnosis and in that way it was possible to establish if they were alive. Mortality rate was 9% and this was relatively high; in study by Gyayi in Uganda the mortality was 7% and other similar studies state 4% to 5% [17–20].

### 4.2. Study Limitations

No postmortems were done for the fatalities and for the patients managed nonoperatively; there could not be intraoperative findings to validate EFAST findings.

The low frequency transducer that was used could have missed some positive findings.

Emergency US scanning performed may be operator dependent, though some studies show that FAST can be reliably done by a radiologist and nonradiologist [21].

## 5. Conclusion

EFAST, an emergency ultrasound scanning technique, is highly sensitive and specific assessment modality for torso injury. It should be adopted for routine use in low resource contexts.

## Figures and Tables

**Table 1:** Distribution of characteristics between EFAST and standard of care.

Characteristic	Number *N* = 197	Percentage
Age		
<18	29	14
19–45	158	80
>45	10	5
Gender		
Male	165	84
Female	32	16
Duration from time of injury to admission		
<1 hour	79	40
>1 hour	118	60
Duration of admission to EFAST		
<1 hour	168	85
>1 hour	29	15
Duration of admission to standard of care		
<1 hour	92	47
>1 hour	105	53
Status of patient at study end		
Dead	18	9
Waiting time for EFAST		
1–30 mins	142	72
31–60 mins	17	9
>60 mins	38	19
Duration lapsed before standard of care investigation was performed		
0–30 mins	8	4
31–60 mins	25	13
>60 mins	164	83
Causes of trauma among the patients recruited		
Road traffic crash	127	64.5
Assault	61	31
Fall	6	3
Others	3	2

**Table 2:** Distribution of external injuries.

External injuries	Number *N* = 197	Percentage
Bruises	168	85
No bruises	29	15

Abrasions	135	69
No bruises	62	31

Lacerations	67	30
No lacerations	130	66

Cuts	30	15
No cuts	167	85

Burns	1	1
No burns	196	99

Injury		
Presence of abdominal injuries	40	20
Absence of abdominal injuries	157	80
Presence of head injuries	102	52
Absence of head injuries	95	48
Presence of chest injuries	130	66
Absence of chest injuries	67	34
Presence of back injuries	47	24
Absence of back injuries	150	76
Presence of lower limb injuries	93	47
Absence of lower limb injuries	104	53

**Table 3:** Validity testing of EFAST taking standard of care as the gold standard.

EFAST	Standard of care		—	—	—	—	—
Clinical findings	Presence	Absence	Total	Sensitivity (%)	Specificity (%)	PPV (%)	NPV (%)
Normal	34	5	39				
Abnormal	0	143	143	100	97	87	100

## References

[1] Brohi K, Singh J, Heron M, Coats T (2003). Acute traumatic coagulopathy. *The Journal of Trauma*.

[2] MacLeod JBA, Lynn M, McKenney MG, Cohn SM, Murtha M (2003). Early coagulopathy predicts mortality in trauma. *The Journal of Trauma*.

[3] Maegele M, Lefering R, Yucel N (2007). Early coagulopathy in multiple injury: an analysis from the German Trauma Registry on 8724 patients. *Injury*.

[4] Radwan MM, Abu-Zidan FM (2006). Focussed Assessment Sonograph Trauma (FAST) and CT scan in blunt abdominal trauma: surgeon's perspective. *African Health Sciences*.

[5] Yuliya A (2010). Emergence sonography for trauma protocol. *Trauma*.

[6] Walcher F, Kirschning T, Müller MP (2010). Accuracy of prehospital focused abdominal sonography for trauma after a 1-day hands-on training course. *Emergency Medicine Journal*.

[7] Boulanger BR, McLellan BA, Brenneman FD (1996). Emergent abdominal sonography as a screening test in a new diagnostic algorithm for blunt trauma. *Journal of Trauma*.

[8] Wangoda RN (2001). *The value of diagnostic peritoneal lavage in the initial assessment of Blunt Abdominal Trauma in Mulago [M.S. thesis]*.

[9] Bugeza S (2006). *The pattern of sonographic findings in blunt abdominal trauma at Mulago hospital [M.S. thesis]*.

[10] McGahan M, Richards J, Gillen M (2002). The focused abdominal sonography for trauma pearls and pitfalls. *Journal of Ultrasound in Medicine*.

[11] Brown MA, Casola G, Sirlin CB, Hoyt DB (2001). Importance of evaluating organ parenchyma during screening abdominal ultrasonography after blunt trauma. *Journal of Ultrasound in Medicine*.

[12] Yoshii H, Sato M, Yamamoto S (1998). Usefulness and limitations of ultrasonography in the initial evaluation of blunt abdominal trauma. *Journal of Trauma—Injury, Infection and Critical Care*.

[13] Zhang ZM, Yang JX, Gan JX, Xu SW, You FD, Joang GY (2006). Rapid detection of pneumothorax by ultra sonography in patients with multiple trauma. *Critical Care*.

[14] Rankine JJ, Thomas AN, Fluechter D (2000). Diagnosis of pneumothorax in critically ill adults. *Postgraduate Medical Journal*.

[15] McKenney KL, McKenney MG, Cohn SM (2001). Hemoperitoneum score helps determine need for therapeutic laparotomy. *Journal of Trauma-Injury Infection & Critical Care*.

[16] Kirschning T, Brenner F, Stier M, Weber CF, Walcher F (2009). Pre-hospital emergency sonography of trauma patients. *Anaesthesist*.

[17] Gyayi M (2005). *Systemic inflammatory response syndrome score of trauma of patients at admission as a predictor of mortality and length of stay in Mulago [M.S. thesis]*.

[18] Odubu-Fualal J (1996). *Pattern of multiple trauma at Mulago Hospital [M.S. thesis]*.

[19] Okello CR, Ezati IA, Gakwaya AM (2007). Missed injuries: a Ugandan experience. *Injury*.

[20] Kobusingye OC, Guwatudde D, Owor G, Lett RR (2002). Citywide trauma experience in Kampala, Uganda: a call for intervention. *Injury Prevention*.

[21] Buzzas GR, Kern SJ, Smith RS, Harrison PB, Helmer SD, Reed JA (1998). A comparison of sonographic examinations for trauma performed by surgeons and radiologists. *The Journal of Trauma*.

